# Predicting human epidermal growth factor receptor 2 status of patients with gastric cancer by computed tomography and clinical features

**DOI:** 10.1093/gastro/goae042

**Published:** 2024-05-08

**Authors:** Yin Li, Wei-Gang Dai, Qingyu Lin, Zeyao Wang, Hai Xu, Yuying Chen, Jifei Wang

**Affiliations:** Department of Gastrointestinal Surgery, The First Affiliated Hospital, Sun Yat-sen University, Guangzhou, Guangdong, P. R. China; Department of Gastrointestinal Surgery, The First Affiliated Hospital, Sun Yat-sen University, Guangzhou, Guangdong, P. R. China; Department of Gastrointestinal Surgery, The First Affiliated Hospital, Sun Yat-sen University, Guangzhou, Guangdong, P. R. China; Department of Surgery, HuiYa Hospital of The First Affiliated Hospital, Sun Yat-sen University, Guangzhou, Guangdong, P. R. China; Department of Radiology, The First Affiliated Hospital, Sun Yat-sen University, Guangzhou, Guangdong, P. R. China; Department of Radiology, The First Affiliated Hospital, Sun Yat-sen University, Guangzhou, Guangdong, P. R. China; Department of Radiology, The First Affiliated Hospital, Sun Yat-sen University, Guangzhou, Guangdong, P. R. China

**Keywords:** gastric cancer, human epidermal growth factor receptor 2, computed tomography, CA125

## Abstract

**Background:**

There have been no studies on predicting human epidermal growth factor receptor 2 (HER2) status in patients with resectable gastric cancer (GC) in the neoadjuvant and perioperative settings. We aimed to investigate the use of preoperative contrast-enhanced computed tomography (CECT) imaging features combined with clinical characteristics for predicting HER2 expression in GC.

**Methods:**

We retrospectively enrolled 301 patients with GC who underwent curative resection and preoperative CECT. HER2 status was confirmed by postoperative immunohistochemical analysis with or without fluorescence *in situ* hybridization. A prediction model was developed using CECT imaging features and clinical characteristics that were independently associated with HER2 status using multivariate logistic regression analysis. Receiver operating characteristic curves were constructed and the performance of the prediction model was evaluated. The bootstrap method was used for internal validation.

**Results:**

Three CECT imaging features and one serum tumor marker were independently associated with HER2 status in GC: enhancement ratio in the arterial phase (odds ratio [OR] = 4.535; 95% confidence interval [CI], 2.220–9.264), intratumoral necrosis (OR = 2.64; 95% CI, 1.180–5.258), tumor margin (OR = 3.773; 95% CI, 1.968–7.235), and cancer antigen 125 (CA125) level (OR = 5.551; 95% CI, 1.361–22.651). A prediction model derived from these variables showed an area under the receiver operating characteristic curve of 0.802 (95% CI, 0.740–0.864) for predicting HER2 status in GC. The established model was stable, and the parameters were accurately estimated.

**Conclusions:**

Enhancement ratio in the arterial phase, intratumoral necrosis, tumor margin, and CA125 levels were independently associated with HER2 status in GC. The prediction model derived from these factors may be used preoperatively to estimate HER2 status in GC and guide clinical treatment.

## Introduction

Gastric cancer (GC) is the fifth most commonly diagnosed malignancy and the third leading cause of cancer-related death worldwide [1]. Human epidermal growth factor receptor 2 (HER2) overexpression occurs in 4.4%–53.4% of patients with GCs [2, 3]. It is currently believed that HER2-positive GC is a unique subtype that requires different treatment strategies. Targeting HER2-positive GCs has been performed in advanced settings for over a decade [4, 5]; promising pathological response rates and survival outcomes have recently been reported for resectable GCs with anti-HER2 blockade in neoadjuvant and perioperative settings [6]. A recent study showed that the addition of trastuzumab to fluorouracil, leucovorin, oxaliplatin, and docetaxel (FLOT) chemotherapy during perioperative treatment of resectable GC resulted in promising histopathological response rates and prolonged disease-free survival, without any unexpected side effect [7]. The PETRARCA study demonstrated that the addition of trastuzumab and pertuzumab to perioperative FLOT chemotherapy in patients with resectable GC resulted in significantly higher pathologic complete response (pCR) rate and pN0 status [8]. The efficacy of targeted therapy is closely related to HER2 expression, and increased levels of HER2 expression predict greater sensitivity to targeted therapy and better overall survival in GC [9]. Therefore, determination of HER2 expression status is essential to select patients with GC who may benefit from anti-HER2 treatment, especially in the neoadjuvant setting, and in advanced-stage GC.

Current HER2 detection methods include immunohistochemical staining (IHC) and fluorescence *in situ* hybridization (FISH) using preoperative biopsy or surgically resected specimens. In both cases, the procedure is invasive, time-consuming, and limited by restricted sample size, potential sampling errors, or complications during the biopsy. Therefore, it is important to establish non-invasive, accurate, and reproducible alternative methods for HER2 status prediction in GC, from which patients with false-negative biopsy results may especially benefit. Accurate, non-invasive prediction may also allow for dynamic detection of HER2 changes during treatment and timely alteration of therapeutic strategies. As a non-invasive method, medical imaging has been widely used in the diagnosis, staging, and prognostic prediction of GC [10]. Previous studies have demonstrated that ^18^F-fluorodeoxyglucose positron emission tomography and the apparent diffusion coefficient value on magnetic resonance imaging can estimate HER2 expression in GC [11, 12]. However, positron emission tomography/computed tomography (CT) examinations are expensive and associated with higher levels of radiation, whereas magnetic resonance examinations require a longer acquisition time and encounter more artifacts.

Multi-slice computed tomography (MSCT) has been widely used for preoperative staging, treatment evaluation, and postoperative follow-up of GC cases [13]. MSCT images can reliably reflect the pathological characteristics of GC, including tumor size, ulcers and necrosis, degree of infiltration, lymph nodes, and distant metastasis [10], especially after enhancement by contrast agent injection, which can better reflect angiogenesis and the microvascular density (MVD) of the tumor dynamically [14]. The association between MSCT features and HER2 expression in GC has been poorly reported [15–17], CT features have not been fully explored, and the findings have been inconsistent. Gao *et al*. [18] developed a dual-energy CT-based nomogram for the evaluation of HER2 expression in GC and demonstrated its potential for HER2 status detection. However, dual-energy CT use is not widespread at present, and the clinical application of a model based on dual-energy CT features may be limited. Moreover, all participants in the aforementioned studies had advanced GC. To the best of our knowledge, there have been no studies on the prediction of HER2 status for resectable GC in the neoadjuvant and perioperative settings.

Therefore, this study aimed to investigate whether a simple and reproducible model could be derived from MSCT imaging and clinical features to predict HER2 status in patients with resectable GC. Such a model may provide a basis for individualized treatment plans for these patients.

## Patients and methods

### Ethics statements

The institutional review board of the First Affiliated Hospital, Sun Yat-sen University (Guangzhou, China) approved this retrospective study, and written informed consent was obtained from all patients. This study was conducted in accordance with the principles of the Declaration of Helsinki.

### Study design and patients

We retrospectively collected data from patients who underwent radical gastrectomy for pathologically confirmed GC at the First Affiliated Hospital, Sun Yat-sen University, between August 2019 and December 2020.

The inclusion criteria were as follows: (i) adult patients (age, ≥18 years); (ii) patients who had not undergone gastrectomy before; (iii) patients who had undergone abdominal contrast-enhanced CT examination within two weeks before surgery; (iv) patients who had received no therapy (chemotherapy, radiotherapy, or surgical treatment) for GC before CT examination; and (v) patients whose GC and HER2 status were confirmed by postoperative pathological examination. Patients were excluded from the study if (i) there were artifacts on the preoperative CT images, (ii) tumor lesions were not visible on CT images, (iii) vascular abnormalities of the portal venous system were present (including portal hypertension, stenosis, thrombus, tumor emboli, and angiodysplasia of the portal vein or superior mesenteric vein), or (iv) malignant tumors were present in other organs. We selected patients with resectable GC because the resected specimens allowed for adequate sampling and provided accurate information on HER2 status. Based on their HER2 expression status, all patients enrolled in this study were divided into HER2-negative and HER2-positive groups.

### Pathological examination

IHC and FISH were used to determine HER2 overexpression and gene amplification, respectively. The HER2 expression profiles of GC were stratified using the Hofmann-modified scoring system for HER2 immunostaining [19]. Scores of 3+ were considered positive for HER2 overexpression, whereas scores of 0 or 1+ were considered negative. An additional FISH test was performed in patients with scores of 2+, for whom the HER2 expression status was uncertain. A ratio of HER2 to centromeric probe 17 of ≥2 was considered positive for HER2 amplification.

### Computed tomography protocol and scanning parameters

All patients were instructed to drink 300–600 mL of 2.5% mannitol solution 30 min before the CT examination to distend the gastrointestinal tract, and an additional 300 mL of the same solution to fully distend the stomach lumen immediately before the examination. Three CT scanners were used in this study. Routine plain scans were obtained first, followed by arterial- and portal-phase-enhanced scans. The enhanced scanning times for both phases adopted a fixed delay time. The reconstructed slice thickness was 1 mm. Details of the CT scan parameters are presented in Table 1.

**Table 1.** Details of the CT scanners and scan parameters

Parameter	CT scanner		
	Toshiba Aquilion VISION	Toshiba Aquilion PRIME	Toshiba Aquilion
Tube voltage, kV	120	120	120
Tube current, mA	Automatic	Automatic	250
Rotation time, s	0.5	0.5	0.5
Detector collimation, mm	80 × 0.5	80 × 0.5	64 × 0.5
CM concentration, mg I/mL	Ultravist, 300	Ultravist, 300	Ultravist, 300
CM dose, mL/kg	1.5	1.5	1.5
Injection flow rate, mL/s	3	3	3
Arterial-phase delay time, s	40	40	37
Portal-phase delay time, s	70	70	70
Pixel size	512 × 512	512 × 512	512 × 512
Slice thickness, mm	1	1	1
Slice interval, mm	0.8	0.8	0.8

CT = computed tomography, CM = contrast material.

### Demographics, laboratory tests, and computed tomography features

Demographic characteristics and laboratory parameters of the participants were retrieved from the clinical database. The demographic characteristics included sex and age. Laboratory parameters included the (i) carcino-embryonic antigen (CEA) (<5 or ≥5 μg/L); (ii) carbohydrate antigen 19–9 (<35 or ≥35 U/mL); (iii) cancer antigen 125 (CA125) (<35 or ≥35 U/mL); (iv) alpha fetal protein (<20 or ≥20 μg/L); and (v) squamous cell carcinoma antigen (<1.5 or ≥1.5 μg/L).

The interpretation of preoperative CT image from all participants was performed independently by two expert radiologists with more than ten years of experience in abdominal radiology who were blinded to patients’ HER2 status. The CT features included involvement of the cardia, well-defined tumor margins, and the presence of ulceration, necrosis, serosal invasion, and positive perigastric lymph nodes (positive lymph nodes were defined as those measuring ≥8 mm in the short axis diameter or internal necrosis) [15, 20], peritoneal metastasis, ascites, and distant metastasis. The maximum diameter of the lesion and maximum tumor thickness perpendicular to the gastric wall were measured on the transverse plane in the unambiguous phase.

We measured the CT values of the lesions and the aorta in the unenhanced, arterial, and portal phases. Avoiding vessels and necrosis, a region of interest was drawn in the area of heterogeneous enhancement. We used the CT value of the abdominal aorta for normalization and calculated the enhancement ratio in the arterial and portal phases: [(CT value in the arterial or portal phase − CT value in the unenhanced phase of the lesion)/corresponding enhanced CT value of abdominal aorta on the same plane]. We also measured the long- and short-axis diameters of the superior mesenteric vein (SMV) on the transverse plane in the portal phase (at the level of the inferior margin of the splenic vein), and the average of the two diameters was recorded as the SMV diameter. The final values for the aforementioned numerical variables were obtained after averaging the individual readings from the two experts in each case. The degree of enhancement in the arterial and portal phases was divided into two categories: lower than the surrounding normal gastric mucosa, and equal to or higher than the surrounding normal gastric mucosa. In case of disagreement between the two radiologists regarding any of the CT features described above, they were re-evaluated until a consensus was reached.

### Statistical analysis

Depending on the variable type and data distribution, univariate analysis was performed using the Student *t*-test, Mann–Whitney *U* test, chi-square test, or Fisher exact test. Variables with a *P* value of ≤0.05 in univariate analyses were subsequently included in multivariate analyses. We assessed the independent risk factors and established a predictive model using logistic regression analyses. Receiver operating characteristic curves were constructed, and the performance of the prediction model was evaluated. The evaluation parameters included the area under the receiver operating characteristic curve (AUC), specificity, and sensitivity. The Brier score was used to evaluate the calibration of the model. The Brier score measures the difference between predicted and actual probabilities, and ranges between 0 and 1. Smaller values correspond to better calibration. Additionally, Nagelkerke *R*^2^ and Hosmer–Lemeshow tests were used to evaluate the goodness of fit of the model. The bootstrap method was used for internal validation. All statistical analyses were performed using SPSS software (version 20.0; IBM Corp., Armonk, NY, USA). Statistical significance was set at *P *<* *0.05.

## Results

### Baseline characteristics

A total of 301 patients with GC (207 men and 94 women, aged 22–89 years) were included in this study. In total, 234 patients were confirmed to be HER2-negative, and 67 patients were HER2-positive. In the univariate analysis, CA125 level, tumor margin, intratumoral necrosis, positive perigastric lymph nodes, enhancement ratio in the arterial and portal phases, SMV diameter, and degree of enhancement in the arterial and portal phases showed statistically significant differences between the two experimental subgroups (Table 2).

**Table 2.** Baseline characteristics of patients in the HER2-negative and HER2-positive groups

Variable	HER2-negative group (*n *=* *234)	HER2-positive group (*n *=* *67)	*P*-value
**Patient demographics**			
Sex, *n* (%)			0.455
Male	158 (67.5)	49 (73.1)	
Female	76 (32.5)	18 (26.9)	
Age, years, mean (SD)	60.59 (12.81)	59.06 (11.76)	0.940
**Laboratory parameters**			
CEA level, *n* (%)			0.057
<5.00 μg/L	193 (82.5)	48 (71.6)	
≥5.00 μg/L	41 (17.5)	19 (28.4)	
CA19-9 level, *n* (%)			0.082
<35.00 U/mL	211 (90.2)	65 (97.0)	
≥35.00 U/mL	23 (9.8)	2 (3.0)	
CA125 level, *n* (%)			0.006
<35.00 U/mL	229 (97.9)	60 (89.6)	
≥35.00 U/mL	5 (2.1)	7 (10.4)	
AFP level, *n* (%)			1.000
<20.00 μg/L	218 (93.2)	63 (94.0)	
≥20.00 μg/L	16 (6.8)	4 (6.0)	
SCC level, *n* (%)			0.407
<1.50 μg/L	108 (46.2)	27 (40.3)	
≥1.50 μg/L	126 (53.8)	40 (59.7)	
**Visual feature**			
Cardia involvement, *n* (%)			0.559
Absent	159 (67.9)	43 (64.2)	
Present	75 (32.1)	24 (35.8)	
Tumor margin, *n* (%)			<0.001
Well defined	172 (73.5)	26 (38.8)	
Poorly defined	62 (26.5)	41 (61.2)	
Ulceration, *n* (%)			0.124
Absent	10 (4.3)	0 (0.0)	
Present	224 (95.7)	67 (100.0)	
Intratumoral necrosis, *n* (%)			<0.001
Absent	195 (83.3)	40 (59.7)	
Present	39 (16.7)	27 (40.3)	
Serosal invasion, *n* (%)			0.053
Absent	133 (56.8)	29 (43.3)	
Present	101 (43.2)	38 (56.7)	
Positive perigastric lymph nodes, *n* (%)			0.025
Absent	107 (45.7)	20 (29.9)	
Present	127 (54.3)	47 (70.1)	
Peritoneal metastasis, *n* (%)			1.000
Absent	230 (98.3)	66 (98.5)	
Present	4 (1.7)	1 (1.5)	
Ascites, *n* (%)			1.000
Absent	229 (97.9)	66 (98.5)	
Present	5 (2.1)	1 (1.5)	
Distant metastasis, *n* (%)			0.072
Absent	228 (97.4)	62 (92.5)	
Present	6 (2.6)	5 (7.5)	
Tumor diameter, mm, median (IQR)	47.65 (29.68)	50.00 (26.60)	0.358
Tumor thickness, mm, median (IQR)	19.70 (8.2)	19.50 (7.2)	0.782
Enhancement ratio in the arterial phase, median (IQR)	0.16 (0.14)	0.24 (0.76)	<0.001
Enhancement ratio in the portal phase, median (IQR)	0.35 (0.16)	0.31 (0.12)	0.023
SMV diameter, mm, median (IQR)	13.25 (3.28)	13.84 (3.60)	0.029
Degree of enhancement in the arterial phase, *n* (%)			0.025
Lower	40 (17.1)	20 (29.9)	
Equal to or higher	194 (82.9)	47 (70.1)	
Degree of enhancement in the portal phase, *n* (%)			0.005
Lower	26 (11.1)	17 (25.4)	
Equal to or higher	208 (88.9)	50 (74.6)	

IQR = interquartile range, HER2 = human epidermal growth factor receptor 2, CEA = carcino-embryonic antigen, CA19–9 = carbohydrate antigen 19-9, CA125 = cancer antigen 125, AFP = alpha foetal protein, SCC = squamous cell carcinoma.

### Prediction model development by CT and clinical features

These factors showing statistically significant differences in univariate analysis were included in the multivariate logistic regression analysis, and the results showed that four of them were independent risk factors for positive HER2 expression: enhancement ratio in the arterial phase (odds ratio [OR], 4.535; *P *<* *0.001), intratumoral necrosis (OR, 2.491; *P *=* *0.017), tumor margin (OR, 3.773; *P *<* *0.001), and CA125 level (OR, 5.551; *P *=* *0.017) (Table 3). The logistic prediction model yielded the following equation: Logit P (the probability of HER2 positive in GC) = −3.547 + 1.512 × enhancement ratio in the arterial phase + 0.913 × intratumoral necrosis (0, absent; 1, present) + 1.328 × tumor margin (0, well-defined; 1, poorly-defined) + 1.714 × CA125 level (0, <35.00 U/mL; 1, ≥35.00 U/mL). The model obtained was statistically significant (χ^2^ = 72.543, *P *<* *0.001).

**Table 3.** Results of multivariate logistic regression analysis of predictors of HER2 status in GC

Variable	Logistic regression			Bootstrap		
	Odds ratio (95% CI)	β (95% CI)	*P*-value	95% CI of odds ratio	95% CI of β	*P*-value
Enhancement ratio in the arterial phase^a^	4.535 (2.220, 9.264)	1.512 (0.798, 2.226)	<0.001	2.382, 15.292	0.829, 2.545	0.002
Intratumoral necrosis						
Absent	Reference	Reference		Reference	Reference	
Present	2.491 (1.180, 5.258)	0.913 (0.165, 1.660)	0.017	1.216, 5.878	0.135, 1.760	0.016
Tumor margin						
Well-defined	Reference	Reference		Reference	Reference	
Poorly defined	3.773 (1.968, 7.235)	1.328 (0.677, 1.979)	<0.001	2.082, 8.171	0.665, 2.215	0.002
CA125 level						
<35.00 U/mL	Reference	Reference		Reference	Reference	
≥35.00 U/mL	5.551 (1.361, 22.651)	1.714 (0.308, 3.120)	0.017	1.375, 37.721	0.219, 3.773	0.008

a Enhancement ratio in the arterial phase was a continuous numerical variable in this study.

CI = confidence interval, HER2 = human epidermal growth factor receptor 2, CA125 = cancer antigen 125; GC = gastric cancer.

### Prediction performance and validation of the model

**Figure 1.**
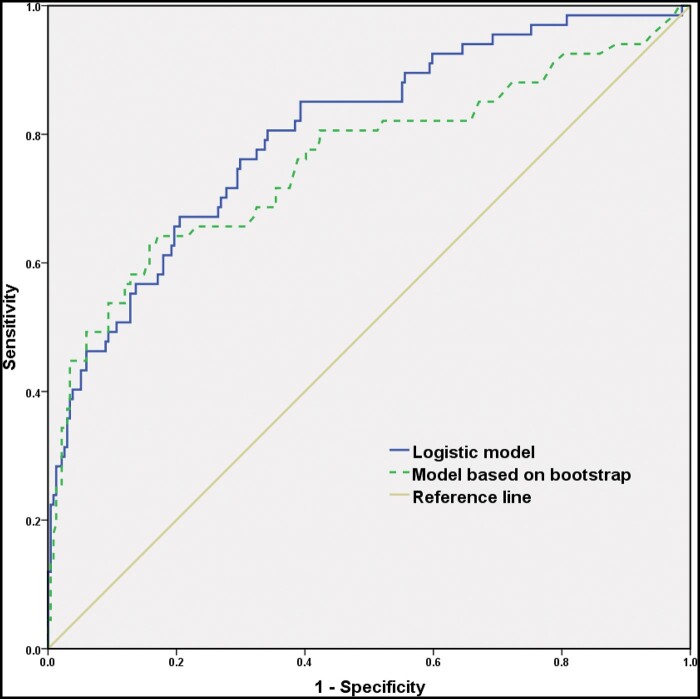
ROC curves of the model based on the logistic (solid line, AUC = 0.802, *P *<* *0.001) and bootstrap method (dashed lines, AUC = 0.758, *P *<* *0.001) for predicting HER2 status in GC. ROC = receiver operating characteristic curve, HER2 = human epidermal growth factor receptor 2, GC = gastric cancer, AUC = area under the curve.

**Figure 2.**
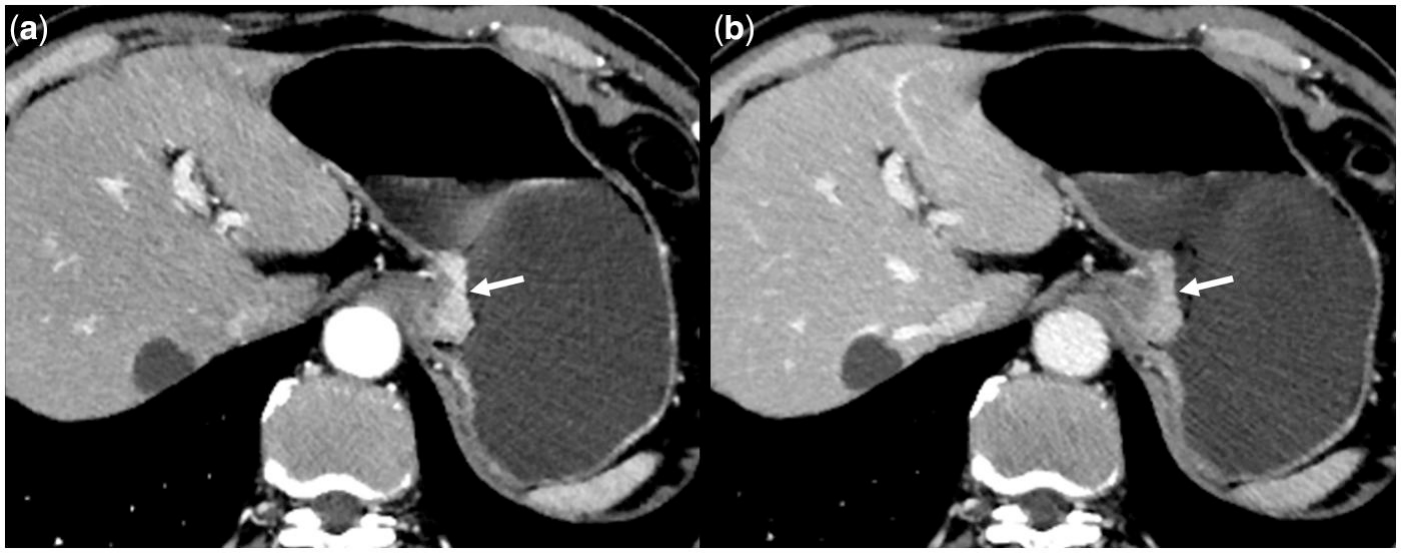
Multi-slice computed tomography scans of a 62-year-old male patient with human epidermal growth factor receptor 2-negative GC. Axial-enhanced arterial phase (A) and portal phase (B) computed tomography images show GC in the lesser curvature of the stomach (arrow), with a well-defined margin, no necrosis, and negative cancer antigen 125. GC = gastric cancer.

**Figure 3.**
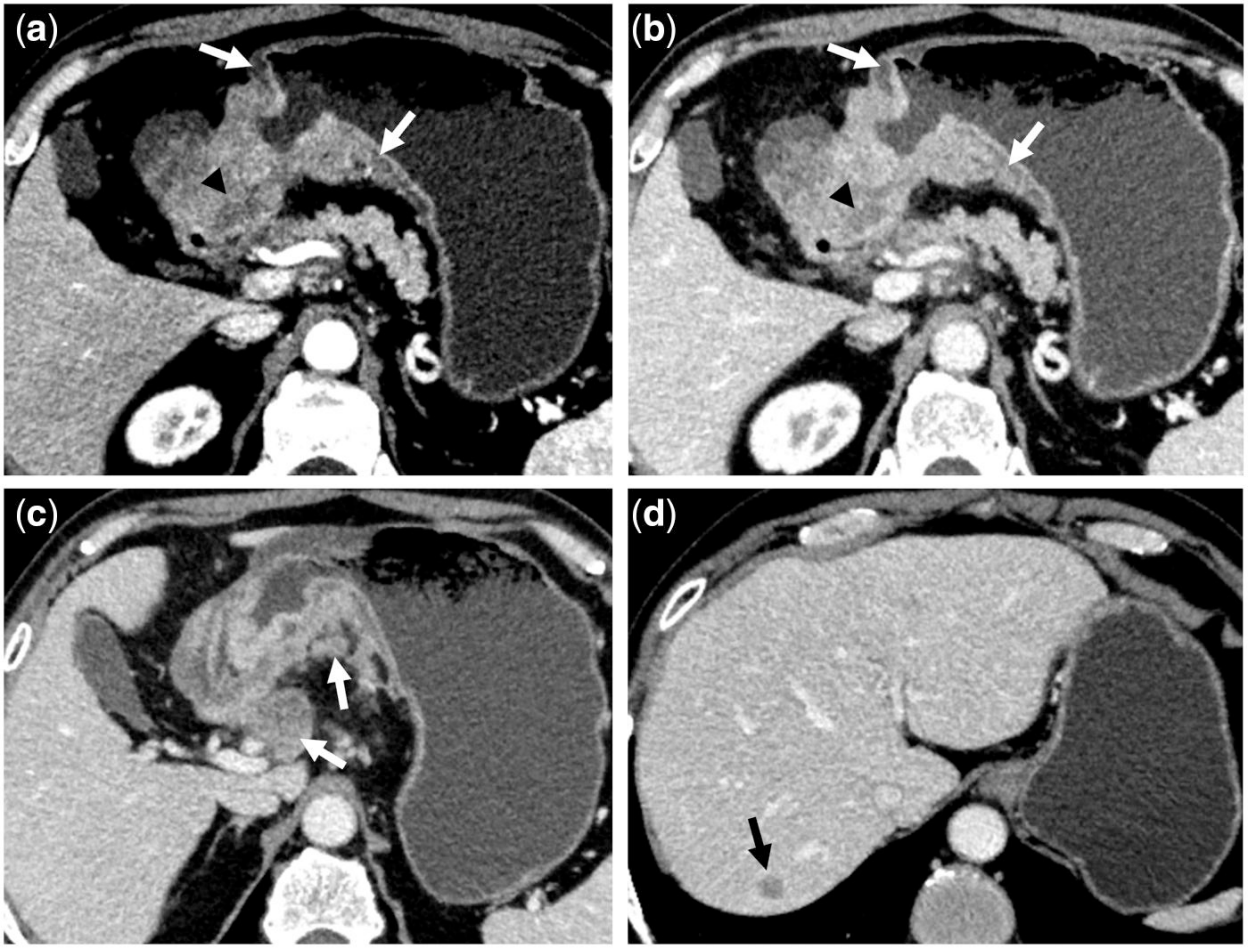
Multi-slice computed tomography scans of a 65-year-old male patient with human epidermal growth factor receptor 2-positive GC. Axial-enhanced arterial phase (A) and portal phase (B) computed tomography images show GC in the antrum and angulus of the stomach, with a poorly defined margin (white arrows in A and B), intratumoral necrosis (black arrowheads in A and B), enlarged perigastric lymph nodes with necrosis (white arrow in C), liver metastases (black arrow in D), and elevated serum cancer antigen 125 level. GC = gastric cancer.

The model correctly classified 83.1% of the study participants. When the Jorden index was considered at its maximum value, the sensitivity, specificity, positive predictive value, and negative predictive value were 67.2%, 79.5%, 71.1%, and 84.8%, respectively. The AUC was 0.802 (95% confidence interval [CI], 0.740–0.864; *P *<* *0.001; Figure 1). The Nagelkerke *R*^2^ value (0.328) for the model and the χ^2^ value (9.490) in the Hosmer–Lemeshow test (*P *=* *0.303) were >0.05, suggesting that the actual and predicted probability distributions were consistent with each other and the model provides a good fit. The Brier score of the established model was 0.1277, which is low, indicating satisfactory calibration results. Internal validation of the bootstrap method based on 1,000 bootstrap samples showed that the model coefficients were stable, with the same risk factors incorporated in the original sample and very similar 95% CIs for the OR values. The bootstrap results showed that the established model was stable and the parameters were estimated accurately. The AUC based on the bootstrap method was 0.758 (95% CI, 0.682–0.834; *P *<* *0.001; Figure 1 and Table 3). HER2-positive and HER2-negative GC samples are shown in Figures 2A, B, and 3A–D, respectively.

## Discussion

In the present study, a predictive model for HER2 status in resectable GC was established using multiphase contrast-enhanced CT image features and serum tumoral markers. We found that enhancement ratio in the arterial phase, intratumoral necrosis, tumor margin, and CA125 level were independent risk factors for positive HER2 expression in GC. The model established based on these four parameters achieved satisfactory predictive efficiency and stability. The accurate prediction of HER2 expression in GC could assist in decision-making regarding chemotherapy regimens, reduce complications, and improve patients’ prognosis.

Studies have shown that contrast-enhanced CT can reflect the blood supply, MVD, and pathological grade of gastric tumors [14, 21, 22]. We found that the ratio of enhancement in the arterial phase was significantly higher in the HER2-positive group than in the HER2-negative group. Wang *et al*. [16] also reported a significant difference in the corrected CT attenuation values in the arterial phase between these two groups. Positive correlations between HER2 expression and MVD in GC [23] and between HER2 and vascular epidermal growth factor (VEGF) expression [24] have been reported. VEGF is an important factor for angiogenesis, whereas MVD reflects new blood vessels at the pathological level, which may explain the rich blood supply observed in tumors from HER2-positive patients, especially in the arterial phase, with a higher enhancement ratio compared to HER2-negative patients. In a study using virtual monochromatic spectral CT, there was a significant correlation between normalized iodine concentration and MVD in gastric adenocarcinoma, and the results showed that normalized iodine concentration could be used to predict angiogenesis [14]. To make the degree of enhancement reflect the blood supply and MVD of the tumor more objectively, abdominal aortic CT attenuation was used to standardize CT attenuation in this study, yielding results that were similar to those obtained with the normalized iodine concentration in spectral CT. Another study showed no significant differences in iodine concentrations in the arterial phase between HER2-positive and negative groups [18]. A possible reason for this is that arterial phase CT images were obtained after an injection delay of 30 s in that study [18], which was in the early arterial phase. However, the enhanced scanning time of the arterial phase in our study adopted fixed delay times of 40 and 37 s according to CT scanners with different detector collimation, which were in the late arterial phase. This suggests that the difference in enhancement may be more significant in the late than in the early arterial phase, which needs to be confirmed by large-sample studies. In the present study, the enhancement ratio in the portal phase was significantly lower in the HER2-positive group than in the HER2-negative group. Similarly, another study showed that the iodine concentration of the tumor in the venous phase (ICVP) and normalized ICVP were significantly lower in HER2-positive GC than in HER2-negative GC [18]. A possible reason for this is that the enhancement ratio in the arterial phase mainly reflects blood supply and MVD, whereas the enhancement ratio in the portal venous phase is an indicator of the balance of blood supply and has a potential role in reflecting the late-phase retention of contrast agents in interstitial spaces [25]. Visually, the proportion of tumor enhancement equal to or higher than that of the normal mucosa in the arterial and portal phases was high in this study, and slightly higher in the HER2-negative group compared to the HER2-positive group. A conflicting result was reported by Lee *et al*. [15], who observed more frequent hyperattenuation in the portal phase in the HER2-positive group than in the HER2-negative group. This may be because subtle differences in enhancement cannot be precisely detected and evaluated when observed under the naked eye.

Our previous study showed that the diameter of the superior rectal vein was significantly increased in patients with rectal cancer carrying the *KRAS* mutation [26]. Inspired by that result, we included the diameter of the gastric drainage vein in the present study. Although the perigastric veins have many variations and are easily invaded by tumors [27, 28], most of them drain to the SMV, which has a constant anatomy and a large diameter that is easy to measure. Therefore, we chose the SMV diameter as the research variable. The results showed that the SMV diameter was significantly larger in the HER2-positive group than in the HER2-negative group. HER2 promotes neovascularization [24]. Tumor-related angiogenesis and destruction of the microcirculation lead to an increased supply of venous blood in the vein, resulting in an increased diameter. However, enlarged SMV was not included in the final prediction model, and therefore its predictive significance needs to be confirmed in further studies with larger sample sizes.

The prognostic role of HER2 in GC is controversial, although some studies have reported that HER2 overexpression is an independent factor for poor prognosis [29–31]. Kim *et al*. [30] found that lymphovascular invasion and lymph node metastasis correlated with HER2 overexpression. According to Zhang *et al*. [32], HER2 overexpression is significantly associated with advanced TNM staging, as well as with tumor progression and poor prognosis in patients with GC. The results of the present study show that the frequencies of poorly defined tumor margins, intratumoral necrosis, and positive perigastric lymph nodes were significantly higher in the HER2-positive group compared to the HER2-negative group. These results are consistent with those of previous studies suggesting that these features represent enhanced aggressiveness. Lee *et al*. [15] also found that metastatic lymph nodes were slightly larger in HER2-positive GCs, although this difference was not statistically significant.

Serum markers can be helpful in detecting recurrence and distant metastases, predicting survival, and monitoring GC after surgery [33]. The correlation between CEA levels and HER2 expression in GC has been previously evaluated, but the conclusions remain controversial [34]. The difference in CEA levels between the two groups in our study was not statistically significant. However, the percentage of serum samples positive for CA125 was significantly higher in the HER2-positive group than in the HER2-negative group. CA125 is useful for detecting peritoneal metastases in GC [33, 35]. The CA125 level was significantly correlated with the degree of peritoneal dissemination. Compared with CT, serum CA125 level has demonstrated superior diagnostic capability for peritoneal metastases [35]. A positive correlation has been found between HER2 overexpression and tumor aggressiveness, and HER2 overexpression is independently associated with poor prognosis in GCs [30]. HER2-positive GCs were more aggressive and more likely to develop peritoneal metastases, whereas hidden peritoneal metastases could not be detected by imaging but elicited elevated CA125 levels.

A previous study reported cases of patients with HER2-positive GC who exhibited marked thickening of the gastric wall on CT imaging [36]. In the present study, lesion thickness and transverse maximum diameter were not significantly different between the two groups, which is consistent with previous findings [15]. Potential reasons for this include the fact that the stomach is an active cavernous organ, and its thickness and diameter are susceptible to changes according to the location and degree of filling. GC is prone to ulceration; thus, the parameter of ulceration s was not statistically significant. Gastroesophageal junction cancer has a higher rate of HER2 expression compared to GC [31], but this was not the case in our study.

This study has several limitations. First, it was a retrospective study, and therefore susceptible to selection bias. Second, this was a single-center study with no independent external validation. Despite the good model stability demonstrated by internal validation using the bootstrap approach, further prospective multi-center studies are required to confirm the robustness of the model. Third, different CT scanners were used. However, the same parameter settings were used in all the scanning equipment, except for the tube current and arterial phase delay time, which were slightly different on one of the scanners. Moreover, we uniformly acquired late arterial phase images (portal veins were visualized but hepatic veins were not), and a slice thickness of 1 mm was adopted. We believe that the CT images used for this study are of a quality acceptable for imaging interpretation and data collection, and that the findings are reliable. However, further prospective studies using standardized and uniform CT protocols are still required to confirm them.

In conclusion, enhancement ratio in the arterial phase, intratumoral necrosis, tumor margin, and CA125 level were independently associated with HER2 status in GC. The prediction model derived from these factors may be useful for estimating HER2 status in GC, which might compensate for the absence of a biopsy specimen, benefit patients with false-negative results in preoperative biopsies, and help guide clinical treatment.

## Authors’ Contributions

J.W. and Y.L. conceived and designed the research, and were responsible for quality control of data. J.W. and W.G.D. performed the statistical analysis. W.G.D., Q.L., Z.W., H.X., and Y.C. collected the CT and clinical data. J.W. and Y.C. performed the interpretation and measurement of the CT images. J.W., Y.L., and Q.L. drafted the manuscript. All the authors edited and made critical revisions to the article. All authors have read and approved the final manuscript.

## Data Availability

The data that support the findings of this study are available from the corresponding authors.
